# Understanding the mental health of youth living with perinatal HIV infection: lessons learned and current challenges

**DOI:** 10.7448/IAS.16.1.18593

**Published:** 2013-06-18

**Authors:** Claude A Mellins, Kathleen M Malee

**Affiliations:** 1 HIV Center for Clinical and Behavioral Studies, New York State Psychiatric Institute and Columbia University, New York, NY, USA; 2 Psychiatry and Behavioral Sciences, Northwestern University Feinberg School of Medicine, Ann & Robert H. Lurie Children's Hospital of Chicago, Chicago, IL, USA

**Keywords:** mental health, psychiatric disorders, emotional and behavioural problems, perinatal HIV infection, adolescence, paediatric HIV

## Abstract

**Introduction:**

Across the globe, children born with perinatal HIV infection (PHIV) are reaching adolescence and young adulthood in large numbers. The majority of research has focused on biomedical outcomes yet there is increasing awareness that long-term survivors with PHIV are at high risk for mental health problems, given genetic, biomedical, familial and environmental risk. This article presents a review of the literature on the mental health functioning of perinatally HIV-infected (PHIV+) adolescents, corresponding risk and protective factors, treatment modalities and critical needs for future interventions and research.

**Methods:**

An extensive review of online databases was conducted. Articles including: (1) PHIV+ youth; (2) age 10 and older; (3) mental health outcomes; and (4) mental health treatment were reviewed. Of 93 articles identified, 38 met inclusion criteria, the vast majority from the United States and Europe.

**Results:**

These studies suggest that PHIV+ youth experience emotional and behavioural problems, including psychiatric disorders, at higher than expected rates, often exceeding those of the general population and other high-risk groups. Yet, the specific role of HIV per se remains unclear, as uninfected youth with HIV exposure or those living in HIV-affected households displayed similar prevalence rates in some studies, higher rates in others and lower rates in still others. Although studies are limited with mixed findings, this review indicates that child-health status, cognitive function, parental health and mental health, stressful life events and neighbourhood disorder have been associated with worse mental health outcomes, while parent–child involvement and communication, and peer, parent and teacher social support have been associated with better function. Few evidence-based interventions exist; CHAMP+, a mental health programme for PHIV+ youth, shows promise across cultures.

**Conclusions:**

This review highlights research limitations that preclude both conclusions and full understanding of aetiology. Conversely, these limitations present opportunities for future research. Many PHIV+ youth experience adequate mental health despite vulnerabilities. However, the focus of research to date highlights the identification of risks rather than positive attributes, which could inform preventive interventions. Development and evaluation of mental health interventions and preventions are urgently needed to optimize mental health, particularly for PHIV+ youth growing up in low-and-middle income countries.

## Introduction

With widespread use of highly active antiretroviral therapy (HAART), children born with perinatal HIV infection (PHIV +) are reaching adolescence and young adulthood in large numbers, such that paediatric HIV is an adolescent epidemic in many parts of the world [1, 2]. Adolescents coping with HIV since birth share stressors experienced by youth with other chronic illnesses, including on-going medical treatment, hospitalizations, exposure to pain and sheltered life experiences [3–5]. They also face a host of unique issues related to the psychosocial impact of HIV, a highly stigmatized and transmittable illness that may make transition through adolescence difficult. Until recently, research on this developmental period for PHIV+ youth has been limited, with focus primarily on biomedical outcomes, adherence to antiretroviral therapy (ART) and prevention of HIV transmission to others. Yet, there is increasing awareness that long-term survivors with PHIV+ are at high risk for mental health problems given exposure to genetic, biomedical, familial and environmental factors [6, 7]. Since HAART was not routinely available to children in the United States (US) until 1998, and until much later for many low-to-middle-income countries (LMIC), many PHIV+ adolescents were exposed to years of sub-optimal treatment and the possibility of active neurotropic and neuroinflammatory HIV disease [8]. Significant and subtle neurocognitive deficits have been observed in PHIV+ children affecting their school achievement, relationships and autonomy [9–11]. HIV may affect subcortical white matter and frontostriatal systems involved in the regulation of emotion and behaviour [12, 13], further placing youth at risk for mental health problems during adolescence [7]. For youth exposed to early severe HIV disease, psychosocial ramifications of hospitalizations, potential mortality, missed school and social opportunities, and delayed puberty, are significant. With age, the impact of these experiences and residual deficits, even in the presence of reconstituted immune systems, may mildly or profoundly limit PHIV+ youth's ability to successfully complete high school, find employment, have relationships and function independently, all of which may have a reciprocal influence on mental health [14].

Multiple family and contextual factors may influence mental health in PHIV+ youth. In many parts of the world, the majority of PHIV+ youth are from ethnic-minority families, living in impoverished, limited-resource communities affected by violence, substance abuse and neighbourhood disintegration [1, 2, 15–17]. These circumstances present challenges for any youth population, but particularly those growing up with a stigmatized health condition [14]. Confronting HIV stigma and managing disclosure of HIV status to others may significantly impact mental health function [18, 19]. Furthermore, many PHIV+ youth live with single parents and/or have experienced multiple caretaking transitions due to parental illness or death [20, 21]. Loss of a parent is one of the most significant stressors linked to poor mental health outcomes [22, 23].

Parental psychiatric and substance abuse disorders are additional risk factors for mental health problems in many PHIV+ youth [24–26]. The potential heritability of these disorders, possible intrauterine exposure to illicit drugs and alcohol, and the stressful family and social environments associated with these disorders can contribute to poor child outcomes [27]. The erosion of parenting capacities that often accompanies illness such as HIV, mental illness, or substance use can be devastating to youth mental health [28].

**Table 1:** Mental health studies of PHIV+ youth

1^st^ Author Ref.	Population Description	Location and Study Type	Mental Health Measures	Mental Health Findings
Bacha (1999) [100]	N=5 PHIV+ youth Age range: 9–12 yrs 3 females, 2 males 2 African-American, 1 Latino, 2 White	Florida pediatric infectious disease clinic, US Pilot study of psycho-educational mental health group	No formal assessment	Caregiver (Cg) and youth reported satisfaction with the group program but no mental health findings reported
Battles and Weiner (2002) [93]	N=80 Cg/youth dyads at time 1(39% PHIV+, 61% transfusion); 55 dyads at time 3 Mean age= 12 yrs at time 1, 13 yrs at time 2, 14 yrs at time 3 Time 1: 56% male Time 1: 72% White, 14% African-American, 7% Hispanic 100% disclosed	National Cancer Institute (NCI), Maryland, US Descriptive longitudinal study	Youth: no mental health assessment; only social support and self-esteem Cg: Child Behavior Checklist (CBCL) Medical chart data-5 yrs post time 1 on psychiatric diagnoses/ hospitalizations, suicidal ideation/ attempts	Youth social support significantly associated with better CBCL scores on withdrawal, anxiety, depression, delinquent, aggression and social problems 5 yr chart data on mental health: a) 3–17 yr olds: 32% anxiety, 45% depression, 13% suicidal ideation, 8% psychiatric hospitalization b) 18+ yr old group: 26% anxiety, 30% depression, 15% suicidal ideation, 4% psych. hospitalization c) loss of a parent associated with depression dx
Bomba (2010) [68]	N=54 (27 PHIV+) 27 HIV− youth=age/gendermatched convenience sample PHIV+ Age range: 5–18 yrs PHIV+: 52% female	Pediatrics departmentUniversity of Brescia, Italy Cross-sectionaldescriptive study	Youth and Cg: PediatricQuality of Life Inventory(PedsQOL) with mental health questions Cg: CBCL Medical chart review of HIV RNA Viral Load (VL)	PHIV+ youth vs HIV− youth had worse scores on: a) overall quality of life, school functioning, and psychosocial health (PedsQOL); b) CBCL internalizing and total problems, but not externalizing scale; and c) withdrawal, anxiety, social, thought, attention and delinquent behavior problem subscales of CBCL VL associated with CBCL delinquent behavior scale Youth not living with bio parent had better CBCL total competence scale
Chernoff (2009) [51]	N=575 (319 PHIV+) Comparison youth: either PHEU or HIV− youth living with HIV+ person (HIV-A) Age range: 6–17 yrs PHIV+=50% male 54% African-American, non-Hispanic, 32% Hispanic	29 US sites International Maternal Pediatric Adolescent AIDS Clinical Trials Group (IMPAACT) 1055 2 yr longitudinal study Baseline analysis	Youth: Child Inventory-4(CI-4)/Youth Inventory-4R (YI-4R) Cg: Child and AdolescentSymptom Inventory-4Revised (CASI-4R); lifeevents and treatment	23% of PHIV+ and 12% of HIV− youth received psychiatric medications PHIV+ youth 2 times as likely to receive stimulants and more than 4 times as likely to receive antidepressants 27% PHIV+ and 17% HIV− youth had behavioral treatments
Elkington (2011) [66]	545 Cg/youth dyads (196 PHIV+, 229 PHEU/HIV-A, 120 HIV− youth) Age range: 9–16 yrs 50% male 50% African-American	NYC hospitals, US. 2 studies: a) CASAH: Child and Adolescent Self-awareness and Health Study; b) Youth with and without HIV+ Mothers Baseline data analysis	Youth: Children's Depression Inventory(CDI); State Trait AnxietyIndex- Child (STAI-C) Cg: CBCL on child;Beck Depression Inventory (BDI) and STAI for self	Mean scores on CBCL within normal range for all groups PHIV+ youth twice as likely to have CDI scores within clinical range than PHEU/HIV-A or HIV− youth Better youth mental health associated with having an HIV+ caregiver
Elliott-DeSorbo (2009) [64]	N=55 PHIV+ youth Age range: 8–17 yrs 55% male 46% African-American; 44% Caucasian	Multiple sites in US Participants from ARV treatment protocols at medical research facilities Cross-sectional study	Youth and Cg: Behavior Assessment System for Children (BASC) Cg: Stressful Events (SLE) Medical Chart data at study visits: CD4 and VL	Means on BASC depression and anxiety scales within normal limits School-related SLE were most common (44%) and predictive of youth report of depression No association of Total # SLE with BASC Disclosure to others associated with youth anxiety
Ellis (2006) [104]	N=19 PHIV+ youth Age range: 2–16 yrs, mean=11 62% male 84% African-American	Children's Hospital of Michigan, US Pilot of Multisystemic therapy (MST)	Retrospective chart review No mental health measures Only health (VL) and adherence assessments	Mental health outcomes not examined even though MST=mental health intervention Statistically significant change in VL from study referral to treatment termination
Fielden (2006) [86]	N=32 (10 PHIV+ youth, 11 caregivers and 11 providers) Youth: Age range: 9–16 yrs Youth: 50% male Youth: 20% Caucasian, 30% of color, 50% aboriginal	British Columbia, Canada Qualitative cross-sectional case study	Data collected through 4 focus groups and 7 in-depth interviews, using semi-structured interview scripts	Participants raised issues concerning: a) mental health, including youth's emotions, bereavement, feeling ‘normal’, security, stability, self-esteem; b) social stigma of HIV; and c) sexual health
Foster (2012) [65]	N=73 (38 PHIV+, 11 PHEU, 22 HIV− non-exposed, 2 unknown exposure status) Age range: 8–17 yrs 50% male PHIV+: 79% African-American, 11% Latino	Baylor College of Medicine, Texas Children's Hospital and University of Miami Pediatric HIV Research Clinics 12 mos longitudinal study	Cg and youth: BASC-2 Neurocognitive tests Sleep assessments	No significant group differences in BASC-2 scores Pro-inflammatory intracellular cytokine factors associated with increased problems on BASC-2 Sleep efficiency associated with fewer parent-reported problems on BASC-2 and cognitive measures (executive function)
Funck-Brentano (2005) [102]	N=30 (25 PHIV+, 5 HIV+ transfusion) Age range: 12–17 yrs 37% male 60% European, 27% African	French Prospective study of psychodynamic oriented support group 10 participants vs 20 who refused or didn't come	No mental health measures Youth measures: perceived illness and treatment experiences, self esteem Medical chart review on health	No mental health analyses Youth in intervention had better perceptions of illness and treatments at 2 years post baseline Percentage of participants with undetectable VL increased from 30 to 80%, vs. no change in the other groups
Gadow (2010) [50]	N=575 (319 PHIV+), HIV− youth=174 PHEU and 82 HIV-A Age range: 6–17 yrs 50% male 86% African-American or Hispanic	29 US sites IMPAACT 1055 sample Longitudinal study Baseline data analysis	Youth: YI-4R and CI-4 Cg: CASI-4R	Both groups showed higher rates of psychiatric disorders than general population PHIV+ youth less conduct disorder and depression and more somatization disorder than PHEU/HIV-A youth PHIV+ youth most prevalent disorders=12% ADHD and 5% Oppositional Defiant Disorder For 73% of PHIV+ and 74% PHEU/HIV− A youth, disorders did not currently interfere with functioning
Gadow (2012) [53]	N=573 (319 PHIV+, 168PHEU-, 86 HIV-A) Entry age range: 6–17 yrs 51% male (PHIV+ group) 48% male (PHEU/HIV−) 86% African-American or Hispanic	29 US sites IMPAACT 1055 sample Longitudinal study Longitudinal data analysis	Youth: YI-4R and CI-4 Cg: CASI-4R	69% PHIV+ and 70% HIV− met DSM-IV criteria for at least 1 psychiatric disorder at at least one time Depression more common for females and youth whose Cg had at least 1 psychiatric disorder Emerging anxiety≥for females and younger youth
Gaughan (2004) [96]	N=1808 PHIV+ and 1021 PHEU PHIV+ median age=10 yrs PHEU median age= 1 yrs 51% female PHIV+: 57% Black, non-Hispanic, 27% Hispanic, 14% White	Multiple sites in US Pediatric AIDS Clinical Trials Group 219C (PACTG219C) Prospective cohort study	Data from PACTG219C database on psychiatric hospitalizations between 2000–2002	All children with psychiatric hospitalization were PHIV+ (n=32); median age at hospitalization=11 yrs Primary reasons: depression (n=16), behavioral disorders (n=8), and suicidal ideation/attempt (n=6) Knowledge of HIV status and experiences of significant life event associated with increased risk of psychiatric hospitalization
Kang (2011) [85]	N=325 (196 PHIV+; 129 PHEU) Age range: 9–16 yrs 50% male 46% African-American, 39% Latino	4 NYC hospitals, US CASAH Longitudinal study	Youth: CDI; STAI-C Youth also completed measures on neighborhood disorder, stressful life events (SLE), problem solving, religiosity Cross-sectional data analysis (baseline)	More neighborhood stress associated with more depression and anxiety for both groups SLE mediated relationship between exposure to neighborhood disorder and mental health No significant differences by HIV status No significant interaction effect between religiosity or problem solving and neighborhood stress on either anxiety/depression
Kapetanovic (2009) [99]	N=236 PHIV+ in short-term and 198 in long-term analyses Entry age range: 3–18 yrs 71% male 11% White, 58% African-American, 29% Hispanic	80 US sites PACTG219C Longitudinal study of participants prescribed second generation anti-psychotics (SGA) vs. matched controls	No mental health measures described Cg report and medical review of psychiatric diagnosis Clinical exam of youth: Body Mass Index (BMI)	No mental health findings reported Association of SGAs (particularly Risperidone) with both short- and long-term changes in BMI Participants receiving both protease inhibitors (PIs) and SGAs showed especially large BMI increases
Kapetanovic (2011) [54]	N=197 PHIV+ youth Entry age range: 13–24 years 56% female 51% Black non-Hispanic, 44% Hispanic 100% disclosed	22 US sites Longitudinal Epidemiologic Study to Gain Insight into HIV/AIDS Children and Youth (LEGACY)	Medical charts for mental health diagnoses (ICD-9) Medical chart data on medication adherence and substance abuse (2001–2006)	55% PHIV+ had at least 1 psychiatric diagnosis, primarily mood (25%) and disruptive (15%) disorders 9% substance abuse disorder Odds of having at least 1 of 3 risky behaviors (ART non-adherence, substance use, sex) were greater among youth with a psychiatric diagnosis
Kmita (2002) [101]	N=30 (17 PHIV+ and 12 HIV-A youth, 1 HIV+ youth infected through transfusion) Age range: 2–15 years Parents of 80% of youth were former drug users None of the children had been told diagnosis by family	Warsaw, Poland Two settings: 1) an outpatient clinic 2) a therapeutic camp for families Psychosocial strategies (individual, family, group) described	Qualitative analysis of audiotapes of sessions	Themes raised: disclosure, stigma in schools, death of parent, multiple losses, child development, and ART problems Therapeutic interventions focused on negative emotions and positive coping Interventions involving both cgs and youth in collaboration with providers were most effective Interventions at clinic and therapeutic camp were considered effective
Lee (2011) [72]	N=219 (54 PHIV+; 165 HIV−) Age≥13 years 47% male 100% PHIV+ disclosed	Thailand hospital and public schools 1:3 Case vs. control, cross-sectional data analysis	Youth: Thai Children's Depression Inventory (CDI) Youth reported on use of substances and sex behavior	PHIV+ youth had lower mean CDI scores and less clinical depression compared to HIV− youth Youth who screened positive for depression were more likely to report sexual intercourse
Lowenthal (2012) [118]	N=692 HIV+ (>90%= PHIV+) Ages 8≤17 50.3% female	Botswana, South Africa clinics in two cities	Cg. Report: Pediatric Symptoms Checklist (PSC)- screening for emotional/ behavior problems	17.3% met symptom cutoff score Virologic failure more common among those with more symptoms of attention/executive dysfunction and depression
Malee (2011) [62]	N=1134 PHIV+ youth Age range: 3–17 yrs 52% female 61% African-American, 24% Hispanic	Over 80 US sites (PACTG 219C) Prospective cohort study Cross-sectional data analysis	Conners’ Parent Rating Scale (CPRS-48) Measures of adherence also included	Youth impairment in CPRS in conduct (14%), learning (22%), somatic (22%), impulsivity-hyperactivity (20%), and hyperactivity (19%) problems Youth with impairment in one or more areas had increased odds of non-adherence In adjusted analysis, odds of non-adherence higher for those with conduct problems or hyperactivity
Malee (2011) [63]	N=416 Cg/youth dyads (295 PHIV+; 121 PHEU) Age range: 7–16 yrs 52% female 81% African-American	15 US sites AMP protocol of Pediatric HIV/AIDS Cohort Study (PHACS) Longitudinal study Cross-sectional data analysis (baseline)	Youth and Cg: BASC-2 Cg: The Parent-Child Relationship Inventory (PCRI) and Cg. psychiatric disorder (CDQ)	Overall mental health problems more likely for PHEU (38%) vs. PHIV+ (25%) youth Both groups: elevated caregiver reported behavior problems, but not youth reported emotional problems Odds of problems associated with: Cg psychiatric disorder, limit-setting problems, and health-related functional limits and youth younger age and lower cognition
Marhefka (2009) [97]	N=164 (60% PHIV+, 40HIV+ behaviorally-infected youth) Age range: 13–21 yrs 52% female 81% Black 62% Heterosexual	5 clinics in NY, Baltimore, and Washington DC Adolescent Impact study Cross-sectional baseline data analysis from intervention trial	Youth report: Achenbach system of empirically based assessment (ASEBA) Medical record review for Psychiatric diagnoses/treatment	31% reported clinically significant levels of internalizing, externalizing and total problem scores, 27% of whom had not received psychiatric care Questioning one's sexual identity associated with more internalizing problems; bisexual identity associated with more externalizing problems No differences by HIV transmission group
Mellins (2006) [76]	N=47 PHIV+ youth and Cg dyads Age range: 9–16 yrs 53% male 83% African-American, 15% Hispanic 77% Disclosed	Pediatric HIV program in NYC, US Cross-sectional study	Youth: Diagnostic Interview Schedule for Children Version IV (DISC-IV); CDI Cg: DISC-IV, CBCL Cg mental health: BDI, STAI, and CDQ (substance use disorder and PTSD only)	55% of PHIV+ youth met criteria for psychiatric disorder on DISC-IV: 40% anxiety, 23% behavioral (21% ADHD), 13% conduct, and 11% ODD CBCL and CDI scores in normative range Cg depression and anxiety associated with worse youth behavioral functioning on CBCL 56% youth had ever been to a therapist
Mellins (2009) [91]	N=320 Cg/youth dyads (193PHIV+ and 127 PHEU)	4 NYC hospitals, US CASAH	Youth: CDI; STAI Youth reports on sexual risk and substance use	No HIV group differences in youth mental health Cg mental health predicted youth mental health
	Age range=9–16 yrs; 50% male 55% African-American, 31% Latino	Longitudinal study Cross-sectional data analysis (baseline)	Cg: BDI, STAI, and parent- child relationship measure	Youth mental health associated with substance use and sexual risk behavior
Mellins (2009) [48]	N=340 Cg/youth dyads (206 PHIV+, 134 PHEU youth) Age range: 9–16 yrs 51% female 54% African-American, 31% Latino	4 NYC hospitals, US CASAH Longitudinal study Cross-sectional data analysis (baseline)	Caregiver and Youth Versions of the DISC-IV Medical charts for PHIV+ youth (CD4+ and VL)	61% of PHIV+ vs. 49% of PHEU met criteria for psychiatric disorder; 33% for multiple disorders PHIV+ youths had higher rates of ADHD and greater use of mental health services than PHEU Older age associated with behavioral disorder ADHD less likely if youth living with bio parent, HIV+ Cg, or Cg with less education HIV health variables and mental health not associated
Mellins (2011) [77]	N=349 Cg/youth dyads (238 PHIV+; 111 PHEU youth) Age range: 10–16 yrs 50% male	15 sites in US and Puerto Rico PHACS Longitudinal study Cross-sectional data analysis	Youth and Cg reports on BASC-2 Youth reports on sexual risk behavior and substance use assessments Youth and Cg reports on adherence to ART Medical charts on VL	43% PHIV+ and 50% of PHEU youth report risks in at least one area (mental health, sex, substance use); 16% PHIV+ and 11% PHEU report>2 risks. Age, but not HIV-status associated with 2 vs 0 risks Among PHIV+youth, detectable VL and living with bio mom associated with having 2 vs 0 risks In PHIV+ most frequent combination of risks was mental health problems and non-adherence (23%)
Mellins (2012) [49]	N=280 youth/Cg dyads (166 PHIV+; 114 PHEU youth) Entry age range: 9–16 yrs 49% male 48% African-American 70% disclosed at baseline, 81% disclosed at follow-up	4 NYC hospitals, US CASAH Longitudinal study Longitudinal data analysis	Youth and Cg: DISC-IV at baseline and at 18 month follow-up (FU) on psych. disorders and substance use disorders (SUD) Medical record review for PHIV+: CD4+ count, VL	69% of PHIV+ and PHEU met criteria for a psychiatric disorder at baseline or FU Among PHIV+ youth, significant decrease in prevalence of any psychiatric disorder (60–44%) Among PHEU, no significant change (57–53%), with significant increase in mood disorders SUD low in both groups, increasing slightly at FU Gender and age differences at baseline, not FU PHIV+ youth had more mental health services at FU
Menon (2007) [70]	N=127 (123 PHIV+) Age range: 11–15 yrs 55% male	Lusaka, Zambia Zambian sample compared to British community sample	Youth and Cg: Strengths and Difficulties Questionnaire (SDQ-Y and SDQ-P)	Zambian PHIV+ youth had greater risk of mental health problems than HIV− British youth Those who reported health problems had higher SDQ-Y scores
	HIV− (age and gender matched peers from British community sample)	Cross-sectional descriptive survey	Youth also reported on feelings about health and peer support group (only the latter was analyzed)	Disclosed PHIV+ youth 2.5 times less likely to score in abnormal range for emotional difficulties, after controlling for age, gender, and ARV treatment
Nachman (2012) [75]	N=319 PHIV+ Age range: 6–17 yrs 51% male 54% African-American, 32%Hispanic	29 US sites IMPAACT 1055 Longitudinal study Cross-sectional data analysis	Youth: YI-4R and CI-4 Cg: CASI-4R	33% any disorder, 18% ADHD, 14% depression, 10% anxiety, 14% disruptive behaviors Little evidence of a relationship between specific ART regimens and severity of psychiatric disorders Inconsistent associations of HIV disease markers and psychiatric symptom severity (e.g. CDC Class C associated with less severe ADHD inattention; higher VL and higher CD4% at baseline associated with>depression)
Nichols (2012) [90]	N=151 PHIV+ Age range: 8 to 18 yrs 54% male 17% white, 54% African-American, 29% Hispanic	38 US sites Data from PACTG P1042s Longitudinal study Cross-sectional data analysis	Youth and Cg report on BASC-2 (SRP and PRS respectively) Adherence data collected	Non-adherence to ART associated with impairment on the BASC-2 SRP: a) Locus of Control scale (youth perceived lack of control over life events or low self-efficacy) and b) Relation to Parents scale (youth reported poor relationships with parents)
Nozyce (2006) [73]	N=274 HIV+ youth Age range: 24 months-17 yrs (median= 7.2 yrs) 47% male 49% African-American, 34% Hispanic	48 US clinical sites (PACTG) Longitudinal study Baseline data analysis	Cg: Conners’ Parent Rating Scale (CPRS) Youth neuropsych measures from medical charts (WISC III for older youth)	On CPRS: 16% conduct, 25% learning, 28% psychosomatic, 19% impulsive-hyperactive, 8% anxiety, and 20% hyperactive problems 52% at least 1 behavioral problem Children>9 years old were more likely to have anxiety problems Lower WISC-III score associated with hyperactivity and behaviors associated with ADHD
Petersen (2010) [87]	N=25 HIV+ youth and 15 Cg Age range: 14–16 yrs 52% male 100% South African	Durban, South Africa Large HIV clinic Qualitative cross-sectional individual interviews	Qualitative analysis of transcripts of in-depth, individual interviews	Youth-reported psychosocial challenges: loss of biological parents, coping with their HIV status, external stigma and discrimination, and disclosure difficulties Cg-reported challenges: disclosure and lack of financial, family and social support Medication, HIV information, a future orientation, and social support identified as important for coping and general well-being of adolescents
Puthanakit (2012) [71]	N=603 (284 PHIV+, 155 PHEU, and 164 HIV−) Age: 1–12 years 58% female 60% Thai	9 sites in Thailand and sites in Cambodia Children with HIV Early Antiretroviral Therapy (CHER) study	Cg report: CBCL Medical chart data on CD4 and whether youth in early or deferred ART treatment arm	Compared to the HIV− controls, PHIV+ youth had higher total and externalizing problem scores PHIV+ youth more likely to meet borderline-clinical cutoff on CBCL compared to control groups
Santamaria (2011) [18]	N=196 PHIV+ Cg/youth dyads Age range: 9–16 years 50% male 58% African-American, 42% Hispanic 70% Disclosed	4 NYC Hospitals, US CASAH Longitudinal study Baseline data analysis	Youth: CDI, STAI-C Cg: CBCL-P Youth reports on HIV stigma and disclosure	Disclosed youth significantly less anxious than non-disclosed youth Disclosure not related to any other mental health outcomes (CDI or CBCL)
Serchuck (2010) [74]	N=576 (320 PHIV+, 256 HIV−) Age range: 6–17 yrs 49% male 49% Black, 36% Hispanic	29 US sites IMPAACT 1055 Cross-sectional study	Youth and Cg:SI-4 Youth and Cg Reports of pain	For PHIV+ only: youth- reported pain associated with higher severity of generalized anxiety, major depression, and dysthymia PHIV+ had more reports of pain than HIV− youth
Sirois (2009) [98]	N=2251 PHIV+ youth 215 have prescriptions for ADHD medications, 2036 without prescriptions Entry age range: 3–19 yrs 53% female 59% African-American, 26% Hispanic	80 US sites PACTG, P219C Longitudinal observational study	Only examined use of commonly prescribed ADHD medications Height and weight measurements	Youth who were prescribed stimulant medications were similar in height and weight growth velocities to general population and to those without stimulant medications Youth who were prescribed non-stimulant medications had height and weight growth similar to general population but slower than HIV+ youth without prescriptions for ADHD; also had diverse neurological and psychiatric diagnoses that could impact growth
Williams (2010) [89]	N=299 (196 PHIV+, 103 PHEU/HIV-A) Entry age range: 6–17 yrs Age range for paper: 12–18 yrs 50% female Race: 46% Black, non-Hispanic 38% Hispanic	29 US sites IMPAACT 1055 Longitudinal study Cross sectional data analysis (baseline)	Youth and Cg Symptom Inventory instruments (YI-4 and CASI-4R) Substance use self-reports	20% met criteria for ADHD, 12% conduct disorder (CD), 15% ODD, and 11% either major depression or dysthymia At entry, 14% reported substance use ADHD, major depression/dysthymia, ODD, and CD diagnosis each associated with greater substance use Link between psychiatric symptoms and substance use did not differ by HIV status
Wood (2009) [55]	N=81 PHIV+ Age range: 13–17 yrs 47% female 72% African-American	Children's Hospital of Philadelphia, PA, US Retrospective cohort study of youth with chart data	Cg: CPRS Psychiatric diagnosis via medical record review	48% with psychiatric illness; 19% with multiple psychiatric comorbidities Lifetime prevalence of disorders: 31% mood disorder, 9% psychotic disorder, 18% ADHD, 14% other behavioral disorders 32% ever received psychotropic medications 16% lifetime history of psychiatric hospitalization Significant association between class C diagnosis and: history of psychiatric illness; multiple diagnoses; mood disorder; psychotic disorder; psychotropic medication use; and psychiatric hospitalization No association between class C status and diagnosis with ADHD or behavioral disorder

**Table 2:** Publications from the major US cohort studies of PHIV+ youth

	Population	Measures	Disorder	Prevalence
**PACTG**				
Nozyce, 2006 [73]	274 PHIV+ 2–17 years (yrs)	CPRS Parent Report	Behavioral Problems	52% with one or more; 16% Conduct Prob., 25% Learning Prob., 19% Impulsiv-Hyper., 8% Anxiety; 20% Hyperactivity
Malee, 2011 [62]	1134 PHIV+ 3–17 yrs	CPRS Parent Report	Behavioral Problems	14% Conduct Prob., 22% Learning Prob., 20% Impulsive-Hyper; 19% Hyperactivity
**CASAH**				
Mellins, 2009 [48]	206 PHIV+, 134 PHEU 9–16 yrs	DISC-IV-youth and caregiver	Psychiatric Disorders (DSM-IV)	PHIV+=61%; PHEU=49% with at least one disorder
Mellins, 2012 [49]	166 PHIV+, 114 PHEU	DISC-IV at entry and 18 mos. follow-up – youth and caregiver	Psychiatric Disorders (DSM-IV)	PHIV+=60% at entry; 44% at FU PHEU=57% at entry; 53% at FU Anxiety disorders were most prevalent at both time points
**CASAH + Risk and Resilience (study of uninfected youth)**				
Elkington, 2011 [66]	196 PHIV+, 129 PHEU, 220 HIV− 9–16 yrs	CDI, STAI-C -youth CBCL-caregiver	Depression, Anxiety; Behavioral Problems	PHIV+=higher likelihood of depression
**LEGACY**				
Kapetanovic, 2011 [54]	197 PHIV+ 13–24 yrs	Diagnostic Interview	Mental health Diagnoses (ICD-9)	55% with at least one psychiatric diagnosis; 25% mood disorder, 17% ADHD, 15% disruptive disorder, 9% substance abuse disorder
**IMPAACT**				
Gadow, 2010 [50]	319 PHIV+, 174 PHEU, 82 HIV-A 6–17 yrs	YI-4R and CI-4- Youth; CASI-4R- Caregiver	Psychiatric Disorder	61% PHIV+ and 62% PHEU/HIV-A with at least one disorder
Williams, 2010 [89]	196 PHIV+, 103 HIV− 12–18 yrs	YI-R-Youth; CASI-4R-Caregiver	Psychiatric Disorder; Substance Use	ADHD=20%; CD=12%, ODD =15%; Depression/ Dysthymia=11% Substance use=14%
Gadow, 2012 [53]	319PHIV+, 168 PHEU, 86 HIV− 6–17 yrs	YI-4R and CI-4- Youth; CASI-4R- Caregiver	Psychiatric Disorder	PHIV+=69%; HIV−=70% with at least one disorder at entry or FU
Nachman, 2012 [75]	319 PHIV+	YI-4R and CI-4- Youth; CASI-4R- Caregiver	Psychiatric Disorder	PHIV+=33% with at least one disorder
**PHACS**				
Malee, 2011 [63]	295 PHIV+, 121 PHEU 7–16 yrs	BASC-2-Parent and Youth Report	Emotional/ Behavioral Problems	PHIV+=25%; PHEU=38% at risk or impaired in emotional or behavioral function
Mellins, 2011 [77]	238 PHIV+, 111 PHEU 10–16 yrs	BASC-2-Parent and Youth Report	Emotional/ Behavioral Problems	PHIV+=43% PHEU=50% with risk in mental health, sexual activity, OR substance use

In summary, there are multiple risk factors for mental health problems in PHIV+ youth. In other populations, mental health functioning is among the most significant predictors of health and behavioural outcomes, with increasing evidence of the economic and social costs of mental health problems to society [29]. In countries with limited financial resources, meeting basic living and health needs of youth are likely the highest priority, yet ignoring youth mental health may preclude youth achievement of health, social and economic stability [16, 30, 31]. This article reviews the literature on the mental health functioning of adolescents who were born with HIV, the corresponding risk and protective factors associated with mental health, mental health treatment modalities and critical needs for future interventions and research. Mental health problems are defined broadly to include psychiatric disorders and indicators of more general psychological distress and emotional and behavioural problems.

For context, an understanding of adolescence as a developmental stage is important. Adolescence is marked by the onset of physical and emotional maturation, accompanied by the challenges of adapting to social, emotional and cognitive changes. Adolescence covers a large age range, beginning as young as nine or 10 and lasting up until 18 or older [32, 33]. Brain development continues through the early 20s and includes neural myelination and synaptic pruning responsible for efficient information processing and executive function [34]. Atypical or compromised brain development may increase adolescent risk for poor impulse control, inhibition, and decision-making and associated problems, including violence, aggression, substance abuse, accidents and risky sexual behaviours [35].

Psychosocial issues are prominent as youth progress through adolescence towards adulthood, attempting to develop a sense of self while striving for autonomy. As relationships with parents and peers change, youth may experience stressful challenges, with immature coping skills and/or inadequate resources. Youth who enter adolescence under adverse circumstances may be ill-prepared to effectively cope with normative changes, making this period particularly challenging [32].

For PHIV+ youth, adolescent developmental tasks are accompanied by the challenges of coping with HIV as a stigmatizing, sexually transmittable chronic illness, the management of medical treatment, and adjustment to family loss. Given the significant association of mental health problems with substance use, sexual risk and poor healthcare behaviours in other populations [29, 36], there is a critical need to understand mental health functioning in PHIV+ youth, identify risk and protective factors associated with mental health outcomes, develop treatment models and inform prevention programmes. Although the paediatric HIV epidemic in the United States and other high-resource countries is near eradication, staggering numbers of children in LMIC have been or will be infected with HIV [2]. With increasing access to ART, they will reach adolescence and young adulthood, requiring proven mental health treatment and prevention programmes.

## Methods

### Search strategy

We conducted an extensive review of online databases including MEDLINE, Psychinfo, PubMed, JSTOR and Google Scholar. Key terminology entered into these databases included: mental health, psychiatric/psychological, emotional and behavioural problems, perinatal HIV infection, paediatric HIV and adolescence. Titles, abstracts and methodology of identified articles were reviewed. In addition to the online databases, reference lists of articles included in the search were examined for additional key studies.

#### Inclusion criteria

Articles included in this review reported data on: (1) PHIV+ youth; (2) youth who were 10 years of age and older (younger youth could be in total sample but sample had to include youth who were adolescents); (3) mental health outcomes; and (4) mental health treatment, including psychopharmacology, mental health services or evidence-based interventions. Only English language articles were included, with no exclusion based on country of origin.

#### Exclusion criteria

Articles were excluded if: (1) no participants were PHIV + ; (2) the majority of the study population was less than 10 years; and (3) original research was not reported. Publication year was not a reason for exclusion but given the focus on adolescence, most articles were published within the past decade. A number of papers identified clinical issues for PHIV+ adolescents, primarily as part of larger reviews on the impact of HIV on children and adolescents, including uninfected youth from HIV-affected families (HIV-A), behaviourally-infected youth, and uninfected, un-affected youth (HIV−) at risk for HIV [7, 37, 38] or as part of opinion papers on the full range of psychosocial needs of PHIV+youth [16, 31, 39]. These clinical reports and review articles are not included, but referenced for the purpose of understanding the data. Several studies described psychiatric functioning of HIV+ adolescents and young adults who were primarily behaviourally-infected [40, 41] and these were also excluded. We are unaware of review articles that have focused only on PHIV+ adolescents and mental health as broadly defined here.

Two research assistants and at least one author read all articles for inclusion and exclusion decisions. A total of 93 articles were reviewed, 55 were excluded and 38 were included (see Table 1).

## Results

### Rates of DSM-defined psychiatric disorders (see Tables 1 and 2)

Relatively few studies focused on rates of psychiatric disorders in PHIV+ adolescents. Sharko reviewed eight studies of Diagnostic and Statistical Manual of Mental Disorders (DSM)-defined psychiatric disorders among HIV+ youth aged 4–20 [42], many, but not all [40, 41], of whom were PHIV+ [43–46]. The average prevalence across studies revealed high rates of attention deficit hyperactivity disorders (ADHD; 29%), anxiety disorders (24%) and depression (25%). However, conclusions relevant to the role of perinatal HIV were difficult to determine, given the large age range (4–21 years), frequent failure to distinguish mode of transmission and limited use of comparison groups.

More recent studies have focused on the prevalence of psychiatric disorders among PHIV+ youth who are close to or within the adolescent age range, using well-validated psychiatric assessments based on DSM-IV diagnoses or medical chart reviews. In the Child and Adolescent Self-Awareness and Health (CASAH) study, a large longitudinal cohort study of PHIV+ youth recruited at age 9–16 from four New York City (NYC) hospitals, a high prevalence of any non-substance use psychiatric disorders (61%) was identified using the Diagnostic Interview Schedule for Children (DISC-IV) [47, 48]. Rates exceeded those of a comparison group of 134 perinatally HIV-exposed but uninfected (PHEU) youth. The most prevalent disorders were anxiety (46%) and behavioural (25%) disorders; mood disorders (e.g., depression, mania, cyclothymia) were less prevalent (7%). The rates of most individual disorders were similar in both groups, although ADHD was more prevalent among PHIV+ (18%) than PHEU (8%) youth. By the 18-month follow-up, rates of any disorder in PHIV+ youth decreased substantively to 44%, while rates among PHEU remained constant [49]. Rates of disorder over time were high, with 69% of youth in both groups meeting criteria for a disorder at either baseline or follow-up; one-third of youth reported comorbid disorders.

Similar results were observed in the International Maternal Pediatric Adolescent AIDS Clinical Trials Group (IMPAACT) 1055 study [50, 51] using the Child and Adolescent Symptom Inventory-4R (CASI-4R) [52]. PHIV+ youth and a comparison group of PHEU youth or HIV− youth with an HIV+ family member (HIV-A), aged 6–17, were recruited from 29 US sites. Psychiatric disorders were identified among 60% of PHIV+ and 62% of PHEU/HIV-A youth at baseline, and 39% and 43%, respectively, at follow-up. Baseline disorders among PHIV+ youth included ADHD (25%), anxiety (24%), disruptive behaviours (22%) and depression (21%). Among those who did not meet diagnostic criteria at entry, comparable numbers of PHIV+ (36%) and PHEU/HIV-A youth (42%) met criteria during follow-up. Similar to CASAH, 69% of PHIV+ and 70% of PHEU/HIV-A youth met criteria for any psychiatric disorder during at least one time point, often resulting in problems with social and academic functioning or use of psychotropic medications (PHIV+=43%; PHEU/HIV-A=37%) [53].

Several studies examined psychiatric disorders through medical chart review with similar findings [54, 55]. For example, in the 22 US-site Longitudinal Epidemiologic Study to Gain Insight into HIV/AIDS Children and Youth (LEGACY) study, 55% of 197 PHIV+ youth (13–24 years) had a documented mental health diagnosis, including mood disorders (25%), disruptive behaviour disorders (28%) and ADHD (17%). However, comparison groups were not included in these studies and it is unclear how documented diagnoses were derived.

Thus, the few cohort studies which examined DSM-IV-defined psychiatric disorders, using different measures, revealed similarly high rates that were considerably higher than comparable studies of youth in the general population [56–58]. However, the studies were all US-based and the specific role of PHIV could not be established given inconsistent associations of HIV with mental health [48, 49, 50, 51, 53] or the lack of comparison groups [54, 55].

### Symptoms of psychological distress or emotional and behavioural problems

Many more studies of PHIV+ youth mental health utilized symptoms checklists of youth- or parent-reported emotional and behavioural problems, depression symptoms, or overall psychological distress (see Tables 1 and 2). Methodology, measures and cohorts varied, as did results across studies. For example, three of the largest cohort studies in the United States, Pediatric AIDS Clinical Trials Group (PACTG) 219C, Pediatric HIV/AIDS Cohort Study (PHACS) and CASAH used well-validated but different instruments to describe mental health symptoms, including the Conners’ Parent Rating Scale (CPRS-48) [59], the Behavior Assessment System for Children, 2nd edition (BASC-2) [60] and the Child Behavior Checklist (CBCL) [61].

PHIV+ children and adolescents, aged 3–17, enrolled in PACTG 219C, an observational late outcome study, had higher rates of behavioural impairment on most scales of the CPRS-48, including conduct, learning and psychosomatic problems, impulsivity–hyperactivity and hyperactivity. Behavioural impairment increased with age for impulsive–hyperactive behaviours and learning problems [62]. Among PHIV+ and PHEU youth, aged 7–16, enrolled in PHACS, a US longitudinal study, mental health problems as assessed on the BASC-2 were more prevalent than in the general population, but were more likely among PHEU than PHIV+ youth. Parent-reported behavioural symptoms were elevated for both groups whereas youth-reported emotional symptoms were not [63]. In contrast, several smaller US cross-sectional studies of PHIV+ youth, aged 8–17, reported BASC depression and anxiety scores within the normative range [64, 65].

Analysis of combined data from CASAH (PHIV+ and PHEU youth, aged 9–16) and a NYC cohort study of uninfected youth (aged 10–14), with and without HIV+ mothers, revealed internalizing and externalizing behavioural problems within the normal range on the CBCL, although uninfected youth with uninfected caregivers had higher rates of problems than other groups [66]. However, on a measure of depression (Child Depression Inventory; CDI) [67], scores were more likely to be in the clinical range among PHIV+ youth than among other groups [66].

Other investigations examined emotional and behavioural problems among PHIV+ youth in other countries, including LMIC. In an Italian study comparing 27 PHIV+ youth, aged 5–18, to a group of healthy age- and gender-matched peers (presumably HIV−), PHIV+ youth had significantly higher CBCL total problem and internalizing problem scores [68]. Similarly, 127 PHIV+ youth in Zambia, aged 11–15, had higher rates of total difficulties, emotional symptoms and peer problems on the Strengths and Difficulties Questionnaire (SDQ) [69] compared to a British sample of age- and gender-matched presumably HIV− youth [70]. Among a Thai and Cambodian sample, PHIV+ youth also demonstrated more problems in the clinical range of the externalizing scale of the CBCL than a comparison group of HIV− youth [71]. In contrast, less depression on the CDI was observed among Thai PHIV+ youth than among age- and gender-matched HIV-peers [72].

To summarize, studies that measured mental health symptoms using different checklists across different countries and regions, at different ages, revealed mixed results. Some suggest that PHIV+ youth have higher prevalence rates than normative data or comparison groups, and others indicate normal functioning, lack of differences with comparison groups, or higher rates of emotional and behavioural problems in PHEU or HIV-A or HIV− youth than PHIV+ youth from similar communities. Differences observed across studies may reflect cohort or cross-cultural differences, including variability in timing of HIV diagnosis and variability in access to and duration of ART or mental health services. Differences are also likely related to methodological variations, including different measures with different cut-offs or severity scores, varying sample sizes and age ranges, and different comparison groups and/or insufficient examination of factors that differentiate groups across investigations.

### Correlates of mental health problems

A number of studies identified risk factors for mental health problems among PHIV+ youth. However, with the exception of basic socio-demographic factors (age, gender, maternal HIV-status, type of caregiver), few studies examined the same characteristics and there is considerable variability in findings. In some studies, child age and gender were associated with the presence of emotional and behavioural symptoms [53, 63, 66, 71], with increased depression and anxiety symptoms found among girls [49, 53], more behavioural problems in boys [48], or more mood and behavioural problems in older youth [48, 73], all consistent with studies in the general population. Other studies found mixed or inconsistent gender differences among PHIV+ youth [63], particularly as youth age [48].

Several studies examined the association of cognitive function and HIV disease characteristics with mental health. PHIV+ adolescents with lower cognitive functioning scores were more likely to have mental health problems on the CBCL [71], BASC-2 [63] and CPRS [73] across countries. In addition to cognitive function, experience of pain [74] and HIV disease characteristics, including the history of AIDS defining illness [55, 75], low CD4 percentage [53, 75] and higher HIV RNA viral load [68, 75] were associated with the presence and/or severity of specific problems, including ADHD inattention symptoms, conduct and depression. However, associations were inconsistent across studies, with some finding no mental health association with disease markers [48, 66, 76].

Examination of the role of maternal/caregiver health yielded mixed results. Some studies found that maternal/caregiver HIV+ status was associated with better youth mental health [48, 66] and others with worse youth mental health outcomes [77]. Although several studies of uninfected children of HIV+ mothers (HIV-A) suggest a significant impact of maternal illness and loss on children [78–81], it has proven challenging to disentangle the impact of perinatal and maternal HIV infection from each other among PHIV+ cohorts. However, consistent with the literature on youth from other populations [82, 83], parental/caregiver mental health was associated with PHIV+ youth mental health, particularly depression and anxiety [48, 53, 63, 66] and illness-provoked caregiver functional limitations increased the risk for child mental health problems [63].

Increasing evidence suggests that social and contextual influences, including exposure to poverty, stressful life events and disadvantaged neighbourhoods, are critical predictors of mental health [29, 84]. However, few studies of PHIV+ youth examined contextual factors, although the vast majority of PHIV+ youth across the globe live in impoverished conditions with exposure to stressful and traumatic life events [2]. In one US-based study, exposure to neighbourhood disorder and stressful life events was associated with higher levels of depression and anxiety in PHIV+ and PHEU youth [85]. In another study, although no association between total stressful life events and youth depression and anxiety was found, school-related stressors were significantly associated with youth self-reported depression [64].

Two qualitative studies from Canada and South Africa utilized focus groups and individual interviews to identify psychosocial issues that could impact youth mental health. These included loss of parents and peers, problems developing a healthy sense of identity and sexuality in the context of a stigmatized and transmittable illness, need for autonomy and sense of self-competence, difficulties with peer affiliation, disclosure and social stigma [86, 87]. Few studies of PHIV+ youth examined the association of many of these psychosocial factors with mental health function, with the exception of disclosure. Unfortunately, findings regarding the impact of disclosure are mixed in PHIV+ adolescent studies and often confounded with age [18]. Among youth who know their diagnosis, some studies suggest less [18] and others more [19] anxiety.

Finally, several US-cohort studies revealed significant co-occurrence of mental health problems with other behavioural risks, similar to studies of adolescents in the general population [88]. Mental health problems in PHIV+ youth were associated with substance use [89], non-adherence [54, 62, 90] and sexual risk behaviour [54, 91].

Importantly, few studies in this review focused on protective factors that promote mental health. Among several that examined positive assets, family process variables including stronger caregiver–child relationships and increased caregiver support [64, 92], caregiver limit-setting [63], and parent–child communication and involvement [66] were associated with better mental health. Similarly, increased peer, parent or teacher social support was associated with less anxiety and depression, fewer withdrawal symptoms and fewer behavioural problems [93].

### Mental health interventions for PHIV+ youth

#### Services research

To address the observed mental health problems, many hospitals and community-based organizations across the globe provide psychosocial services for PHIV+ youth. Although a number of case reports and descriptions have been published [17, 94, 95], few service programmes were evaluated for efficacy. Similarly, many of the large US-cohort studies documented the use of mental health treatment among PHIV+ youth, but few examined the impact of this treatment on mental health outcomes. Among PHIV+ and PHEU/HIV-A youth in IMPAACT 1055, 18% of youth received psychotropic medications, and 22% received behavioural treatment, including individual, family and group counselling, behavioural modification, after-school tutoring and psychiatric hospitalization [51]. PHIV+ youth were more likely to receive such treatment, but the mental health impact was not reported. Medical chart data from PACTG 219C indicated that psychiatric hospitalization was more likely for PHIV+ than PHEU youth; however, a significant age confound in this study limited conclusions [96]. Finally, in another study, 27% of youth with clinically elevated behavioural problem scores had not received treatment for identified problems, despite available mental health services [97].

#### Psychopharmacology

Psychopharmacological treatments for mental health problems have been successful with adolescents from other populations. However, few studies examined psychopharmacological approaches in PHIV+ youth, with the exception of PACTG 219C. An examination of stimulant and non-stimulant medication prescribed for PHIV+ youth found slower rates of growth in height and weight for those on non-stimulant medications [98]. In another study, prescribed second-generation antipsychotic (SGA) medications, in general, and risperidone, specifically, were associated with both short- and long-term changes in body mass index (BMI) Z-scores [99]. Neither of these reports evaluated adherence to the prescribed medications or treatment effects on mental health, yet both point to the need for caution and continued pharmacological studies including assessment of impact.

### Evidence-based interventions

We found no evidence-based interventions targeting only mental health outcomes in PHIV+ youth, although several papers described preliminary evaluations of treatment programmes [100–102] and pilot trials of interventions [103–105]. For example, Funck-Brentano and colleagues evaluated a psychodynamic group therapy programme for French PHIV+ adolescents, aged 12–18, who met every 6 weeks for 26 months [102]. Participants acquired HIV either perinatally or through blood transfusions. Outcomes in 10 participants were compared to 10 youth who refused treatment and 10 youth who could not access the clinic. While the intervention was promising in terms of improved viral load and perceptions of health and treatment, mental health was not assessed and groups were not randomly assigned. Two other programme evaluations (Poland and United States) only described qualitative feedback of participants or providers [100, 101].

Multisystemic therapy (MST) [103], originally designed for adolescents with antisocial behaviour, is an intensive family-centred community-based psychotherapy approach. Evidence-based techniques drawn from cognitive behavioural and behavioural family therapy and parent training are utilized. MST was adapted for use as an adherence intervention for non-adherent PHIV+ youth [104]. Psychotherapists delivered MST to 19 participants (mean age = 11 years), 2–3 times a week for approximately 7 months. Although the results were promising in terms of improved health outcomes, the absence of mental health assessment precludes conclusions regarding mental health impact. Moreover, a larger scale randomized controlled trial (RCT) has not been done and MST remains costly and labour-intensive, which may prove prohibitive in LMIC.

Finally, the Collaborative HIV/AIDS Mental Health Program (CHAMP), a family-based intervention originally developed to promote mental health and reduce sexual risk behaviour among inner-city uninfected pre- and early adolescents in the United States [105], was adapted for PHIV+ early adolescents (CHAMP + ) [105, 106]. CHAMP is a 10-session multiple family group programme administered by lay staff to address family variables (parent–child supervision, monitoring, communication, involvement and support), and youth variables (coping, self-esteem, mental health and peer negotiation skills). In multiple clinical trials, families involved in CHAMP consistently demonstrated significant improvements relative to comparison groups in family and youth variables, including mental health [107–109].

CHAMP+ was created for PHIV+ youth to address the above topics, as well as ART adherence [105, 106]. Preliminary evidence from three pilot trials based in the United States, South Africa and Argentina showed promise in promoting family supervision, monitoring and communication, as well as child adherence, self-esteem and mental health. A larger trial is currently being initiated in South Africa [92].

## Discussion

Studies to date suggest that youth born with HIV are at high risk for mental health problems, although HIV infection per se may not be the primary mechanism. There is emerging consensus that the aetiology of psychiatric disorders and other mental health problems is a diagnostic challenge and multifactorial, given the abundance of risks and potential pathways to poor mental health in this population. PHIV+ youth are from vulnerable backgrounds with a constellation of biomedical, genetic, familial and environmental characteristics that have been associated with mental health problems in other populations. Although studies are limited with mixed findings, this review indicates that child HIV and health status are not consistent predictors of mental health problems in the United States or LMIC. Other factors, such as age, worse cognitive function, parental health and mental health, stressful life events and neighbourhood disorder have been associated with worse mental health outcomes in multiple studies, while other factors such as parent–child involvement and communication, and peer, parent and teacher social support have been associated with better function. Unfortunately, this review highlights many gaps and limitations, precluding both firm conclusions and full understanding of aetiology. However, these limitations present opportunities for future mental health research.

The vast majority of studies took place in the United States where the perinatal HIV epidemic is near eradication. There is a clear need for research with PHIV+ youth in sub-Saharan Africa and Asia, where hundreds of thousands of PHIV+ children will reach adolescence in upcoming years [2]. Although many studies have focused on orphans and vulnerable children affected by parental HIV/AIDS, very few studies in Sub-Saharan Africa have focused on the mental health of PHIV+ adolescents and we found none that examined psychiatric disorders. The lack of diagnostic instruments validated for use in this context is likely a significant limitation to extending this work to where the need is most critical. That said, only a few studies in any context examined psychiatric disorders using validated DSM-IV referenced psychiatric interviews, and several used chart reviews with limited information on the professional training of the informant, indicating a global need for studies of psychiatric functioning.

Considerably more studies examined mental health symptoms. However, different checklists were used with different outcomes and scoring systems, making it difficult to compare clinical significance across studies and countries. Not all measures had validated clinical cut-offs. Some studies had relatively small cohorts with large age ranges, including older children and pre-adolescents, with limited power to examine age differences or confounding factors. There is overlap among children and adolescents enrolled in some cohort studies, with multiple papers from the same project, such that results across studies were not necessarily independent (Table 2). The use of different comparison groups (PHEU, HIV-A, HIV− youth) or lack thereof was an additional noteworthy constraint.

In spite of the number of studies identifying mental health problems, and a number of opinion pieces highlighting needs, we identified few evaluations of mental health services or evidence-based mental health treatment programmes targeting PHIV+ adolescents. The identified studies focused either on non-mental health outcomes (e.g. health outcomes or adherence), or a combination of health and mental health outcomes. Further, we found no published RCTs of treatments for specific disorders among PHIV+ youth.

## Recommendations

(1) It is clear that the vast majority of research on PHIV+ adolescents focused on risk, yet in other vulnerable populations of youth, there is increasing attention being paid to “resilience” or “positive youth development” [110–112]. Resilience, typically defined as positive development despite exposure to significant adversity, and positive youth development models that focus on youth strengths regardless of adversity exposure, have been helpful in identifying youth, family and community characteristics which can be utilized in preventive interventions to promote positive psychosocial function. For example, studies in other populations suggest that key strengths amenable to interventions include: (a) at the individual level, youth social, academic and emotional competence, self-regulation, problem-solving skills and adaptive coping; (b) at the family level, parent–child relationships/involvement, family communication and support; and (c) at the contextual level, school and community support systems [110–112].

Resilience models have not been widely used in research with PHIV+ youth [38], yet multiple studies suggest that many PHIV+ youth display signs of resilience despite vulnerability [49, 63, 66]. There is a substantive need for studies of both risk and resilience in PHIV+ youth at different developmental stages and in different cultures so that appropriate preventive interventions can be developed. As yet, it is unclear from the literature whether there is one typical pattern of resilience or whether population-specific risk and protective factors and/or individual level variables are important, and whether there are critical ages that are most amenable to interventions [112]. Equally important are studies grounded in theoretical models of behavioural health that could identify factors amenable to prevention for the development of evidence-based interventions.

(2) Evidence-based mental health treatments for psychiatric disorders have been successful in other populations, including cognitive-behavioural therapies [113], interpersonal psychotherapy [114], dialectical behavioural therapy [115] and family systems approaches [116]. We found no studies of these interventions with PHIV+ adolescents. Research is needed to assess the utility of these interventions for PHIV+ youth, and whether modifications might be necessary to address unique issues such as HIV-related health and neurological complications, and grief-related complications due to loss. As previously demonstrated, mental health interventions are most effective when they are tailored to specific populations and cultures [112, 117].

(3) Given that the majority of PHIV+ adolescents lives or will live in LMIC with limited resources for mental health evaluation and treatment [2], there is an urgent need for larger cohort studies in these contexts using reliable and valid assessment tools that can be used across cultures. Developing such tools should be a priority for future work. Efforts are underway, for example, to refine a mental health screening tool that could be utilized to allocate scarce psychosocial evaluation and support for those most in need [118].

(4) In addition, evidence-based mental health treatment programmes that can be used by community-based workers or lay staff are urgently needed in LMIC, where the dearth of psychiatrists, psychologists and other mental health professionals is significant. In some African countries, healthcare systems are beginning to use a task-shifting or task-sharing approach in which community-based lay counsellors under the supervision of healthcare professionals are providing an increasing number of services, including mental health treatment [119]. A critical strength of CHAMP+ is that it was developed with this professional shortage in mind and can be administered by lay counsellors [92, 109].

(5) PHIV has been described as the prototypical bioneuropsychosocial disease, with risks from the cellular to the behavioural to the social and structural level [37, 92]. As such, the scope of mental health research with PHIV+ youth going forward should exploit findings from behavioural and social sciences but also from genomics and neuroscience, both of which are already shifting our understanding of psychiatric illness in general [120]. New insights may improve our ability to identify early signals of mental illness and have important implications for type, timing and intensity of interventions. For example, results from research with PHIV+ infants and children during the earlier years of the US epidemic could be examined in concert with current investigations of these same aging youth to determine whether such signals exist in this population and to describe their trajectory.

(6) There remains a substantive need for studies that examine multiple factors associated with mental health problems at different stages of childhood, adolescence and young adulthood in PHIV+ youth across contexts to inform preventive interventions. These multi-level investigations necessitate theoretical models and statistical approaches that examine multiple pathways of causality as well as mediating and moderating effects. Moreover, meta-analysis of results of previous and current investigations is likely a critical next step in further clarifying the nature and extent of mental health problems among PHIV+ youth as well. Statistically, this process would help ascertain the commonalities in various prevalence estimates and also clarify conclusions that were difficult to discern.

## Conclusions

The results of this review suggest a high need for mental health treatment programmes for PHIV+ youth as well as mental health-related research, particularly in LMIC. Although research-to-date indicates that adolescence is a risky period for poor behavioural outcomes in vulnerable populations, adolescence has also been conceptualized as a strategic opportunity for healthy development, including mental health. Service and research systems with both a risk and resilience perspective may be most effective in ensuring healthy development for all youth, including those who have grown up with HIV.
